# Enhanced structural connectivity within the motor loop in professional boxers prior to a match

**DOI:** 10.1038/s41598-021-88368-4

**Published:** 2021-04-27

**Authors:** Yuichi Ogino, Hiroaki Kawamichi, Daisuke Takizawa, Sho K. Sugawara, Yuki H. Hamano, Masaki Fukunaga, Keiko Toyoda, Yusuke Watanabe, Osamu Abe, Norihiro Sadato, Shigeru Saito, Shigeru Furui

**Affiliations:** 1 Department of Anesthesiology, Gunma University Graduate School of Medicine, 3-39-15 Maebashi, Gunma, 371-8510 Japan; 2 Department of Anesthesiology, Japanese Red Cross Medical Center, 1-22 Hiroo, Shibuya-ku, Tokyo, 150-8935 Japan; 3 Neural Prosthesis Project, Tokyo Metropolitan Institute of Medical Science, 2-1-6 Kamikitazawa, Setagaya-ku, Tokyo, 156-8506 Japan; 4 Division of Cerebral Integration, Department of System Neuroscience, National Institute for Physiological Sciences, 38 Nishigonaka, Myodaiji, Okazaki, Aichi 444-8585 Japan; 5 Department of Radiology, Graduate School of Medicine, The University of Tokyo, 7-3-1 Hongo, Bunkyo-ku, Tokyo, 113-8655 Japan; 6 Department of Radiology, The Jikei University School of Medicine, 3-28-8 Nishi-Shimbashi, Minato-Ku, Tokyo, 105-864 Japan; 7 Department of Radiology, Graduate School of Medicine, Teikyo University, 2-11-1 Kaga, Itabashi-ku, Tokyo, 173-8605 Japan

**Keywords:** Basal ganglia, Motor cortex, Brain imaging

## Abstract

Professional boxers train to reduce their body mass before a match to refine their body movements. To test the hypothesis that the well-defined movements of boxers are represented within the motor loop (cortico-striatal circuit), we first elucidated the brain structure and functional connectivity specific to boxers and then investigated plasticity in relation to boxing matches. We recruited 21 male boxers 1 month before a match (Time1) and compared them to 22 age-, sex-, and body mass index (BMI)-matched controls. Boxers were longitudinally followed up within 1 week prior to the match (Time2) and 1 month after the match (Time3). The BMIs of boxers significantly decreased at Time2 compared with those at Time1 and Time3. Compared to controls, boxers presented significantly higher gray matter volume in the left putamen, a critical region representing motor skill training. Boxers presented significantly higher functional connectivity than controls between the left primary motor cortex (M1) and left putamen, which is an essential region for establishing well-defined movements. Boxers also showed significantly higher structural connectivity in the same region within the motor loop from Time1 to Time2 than during other periods, which may represent the refined movements of their body induced by training for the match.

## Introduction

Plasticity is an intrinsic property of the human brain^1^ and refers to structural or functional changes (which may trigger each other) that occur in the human brain to adjust to changes in the external or internal environment^2,3^. Recently, because physical training and learning skills alter the human brain structure, there has been a growing interest in the study of the structural and functional plasticity of elite athletes’ brains, which results in exceptional performance through training^4,5^. Brain structure differs among different sports because different motor and cognitive abilities are required for each type of sport^6^. Training also induces structural changes corresponding to the required skill for each sport^7^. For example, after training, jugglers showed increased gray matter volume (GMV) in the mid-temporal cortex, the brain area responsible for analyzing visual movement^8^. However, the jugglers in the study^8^ were non-athlete volunteers, and the training for juggling was not training for a match that would discriminate between “winners” and “losers.” To achieve victory in a match, appropriate and elaborate training is required beforehand^9–11^. From this viewpoint, the neural correlates underlying training before a match should be investigated.

For approximately 1 month before the weigh-in (24 h before the match), professional boxers undergo physical training to reduce their body weight and restrict their oral intake to compete in several pre-designated weight divisions (body mass is used as a proxy for body size)^9–12^. The significance of weight reduction for boxers before a match is described by the Japanese Boxing Commission (JBC), the governing body of Japan’s professional boxing league (https://www.jbc.or.jp/), to refine the accurate and smooth movements by trimming off excess body fat. The “increasing well-defined movement of the body” in boxers can be defined as the smoothness of the sequential motor movements acquired through skill training^13^. For instance, the one-two combination punch (a left jab and a straight punch of the dominant right arm) is a representative sequential motor movement in the training of boxers^14^. The smoothness of the body movement is highly correlated and represented by neuronic activity in the distributed network of the cortico-striatal circuit (also called the “motor loop”). This circuit mainly involves the striatum, thalamus, and motor cortical regions (premotor area, supplementary motor area, and primary motor area)^15,16^. When a motor skill is well learned and acquired, the representation of the motor sequence is distributed within the motor loop^17,18^. The well-defined movement acquired by motor skill training has also been shown to be associated with an increase in myelin, as measured by diffusion-weighted imaging (DWI), suggesting that the skilled movement increases structural connectivity in white matter in addition to improving functional connectivity^19^.

**Figure 1:**
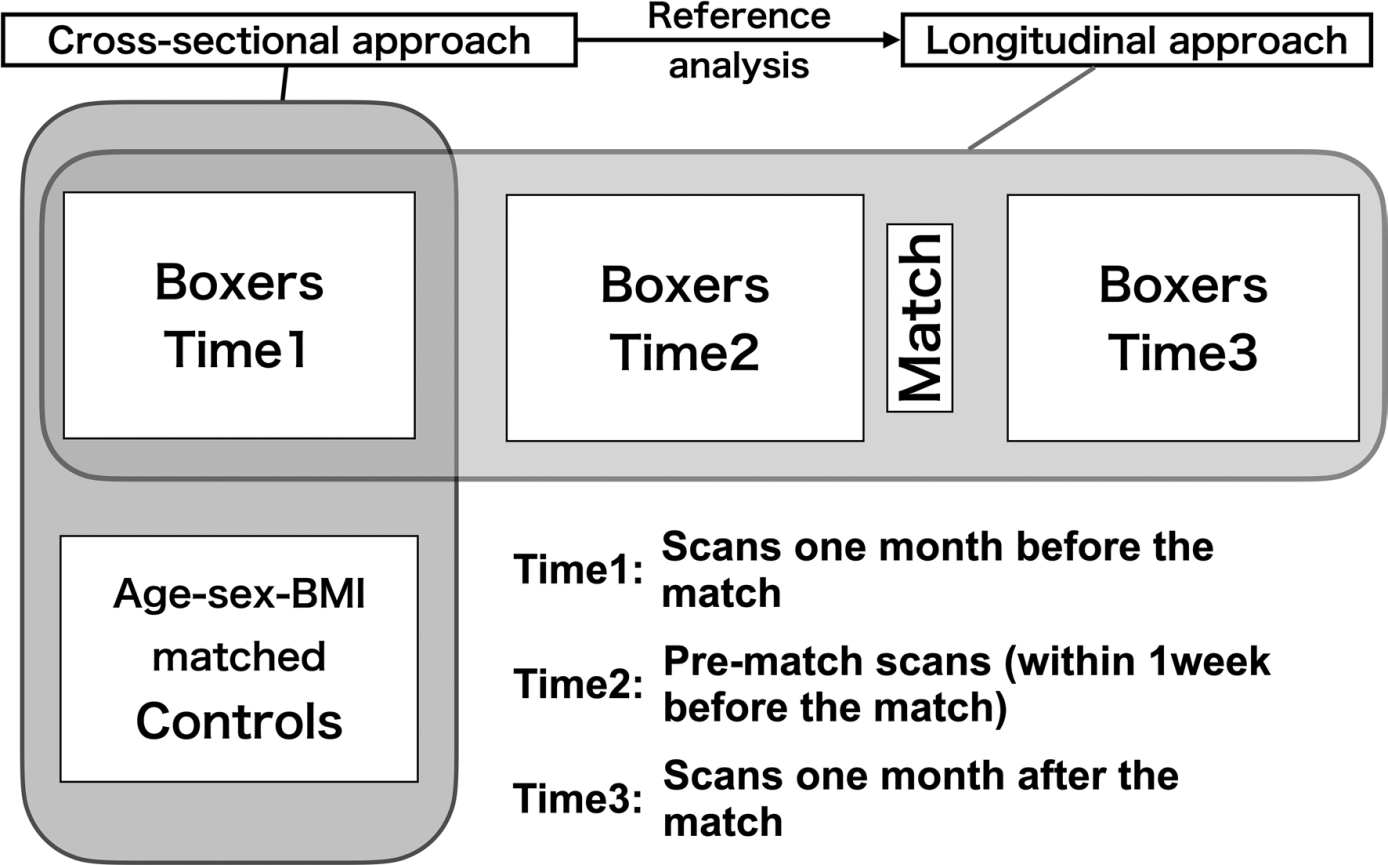
General design. We included 21 licensed male boxers and 22 age-, sex-, and BMI-matched controls. We followed up boxers at time points Time2 and Time3. All imaging statistical thresholds for VBM and rs-fMRI were set to uncorrected p < 0.001 at the voxel level, and FWE-corrected p < 0.05, at the cluster level. FWE, family-wise error; rs-fMRI, resting-state functional magnetic resonance imaging; VBM, voxel-based morphometry.

Thus, we hypothesized that the heightened well-defined movements of the body of boxers before the match would be represented by enhanced functional and structural connectivity within the motor loop. We first elucidated the brain structure specific to boxers. Then, we investigated their functional and structural plasticity around the time of the match. To elucidate the boxer-specific brain structure, voxel-based morphometry (VBM)^20^ was conducted to examine the structural differences between boxers and age-, sex-, and BMI (body mass index: weight [kg/height [m]^2^)-matched non-athlete controls. Based on the results of the VBM analysis, we then conducted seed-based functional connectivity analyses^21^ of resting-state functional magnetic resonance imaging (rs-fMRI) data to elucidate boxers’ specific functional connectivity. We then investigated the brain plasticity of functional and structural connectivity in boxers by measuring the following three time points: 1 month before a match (Time1), within 1 week of the match (Time2), and 1 month after the match (Time3) (Fig. 1). We anticipated that any structure specific to boxers would be represented in the brain regions associated with motor skill training and that boxers’ functional and structural connectivity within the motor loop would be enhanced before the match.

## Results

### BMI and match results

**Figure 2:**
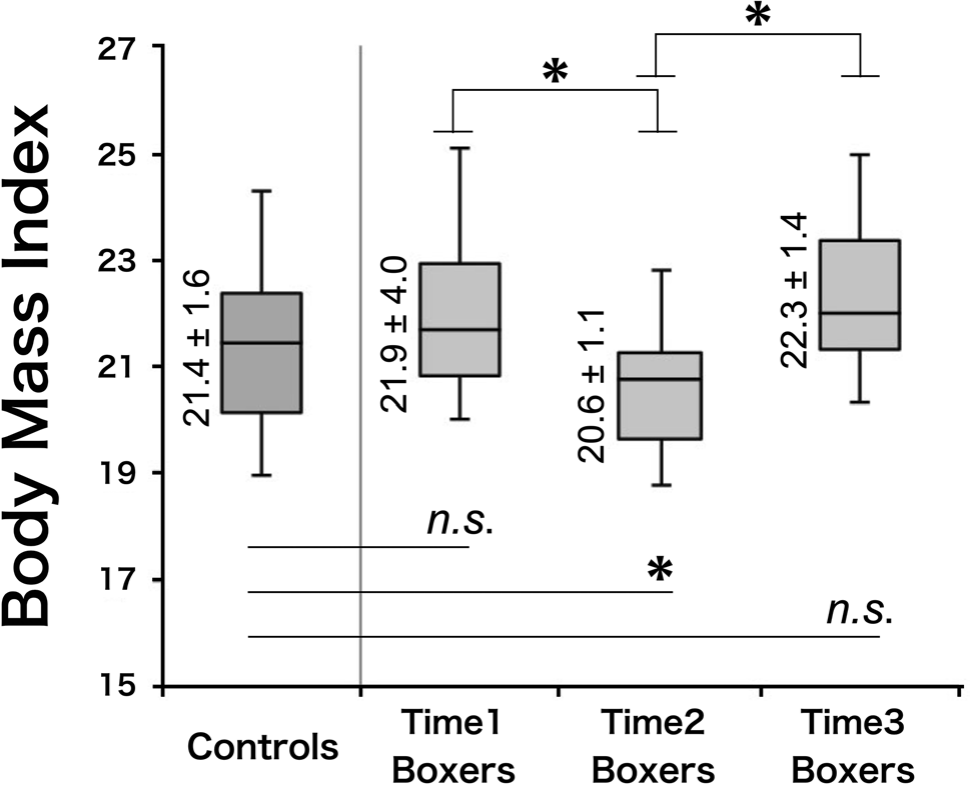
Body mass index of controls and boxers. The boxer BMI at Time2 was significantly decreased [*F*(2, 59) = 10.21, *p* < 0.01] in repeated measures ANOVA. Bonferroni correction was used for the post-hoc analysis. BMI, body mass index; *n.s.*, not significant.

The BMIs of boxers were as follows: 21.9 ± 4.0 at Time1, 20.6 ± 1.1 at Time2, and 22.3 ± 1.4 at Time3, while the BMIs of the controls were 21.4 ± 1.6 (Fig. 2). The BMI decrease in boxers from Time1 to Time2 was − 1.37 ± 0.7. The BMIs of boxers were significantly decreased in Time2 [*F*(2, 59) = 10.21, *p* < 0.01] in repeated measures ANOVA. Bonferroni correction was used for post-hoc analysis. The demographics of the participants are presented in Table 1. A total of 21 match outcomes were as follows: 14 wins, one draw, and six defeats.

**Table 1:** Controls and boxers (Time1, Time2, and Time3).

	Controls	Boxers Time1	Boxers Time2	Boxers Time3
N	22	21		
Age	27.2 ± 3.8	26.7 ± 4.0		
BMI	21.4 ± 1.6	21.9 ± 4.0	20.6 ± 1.1 *	22.3 ± 1.4
Handedness	All right-handed	All right-handed		
Stanford Sleepiness Scale	2.5 ± 1.1	2.8 ± 0.6	2.9 ± 0.8	2.7 ± 1.0

Controls were non-athlete male participants age-, sex-, and BMI-matched with boxers at Time1. The BMI at Time2 was significantly decreased [*F*(2, 59) = 10.21, *p < 0.01] in repeated measures ANOVA. Bonferroni correction was used for the post-hoc analysis.

BMI, body mass index; N, number of participants; Time1, 1 month before the match; Time2, within 1 week before the match; and Time3, 1 month after the match.

### VBM results

**Figure 3:**
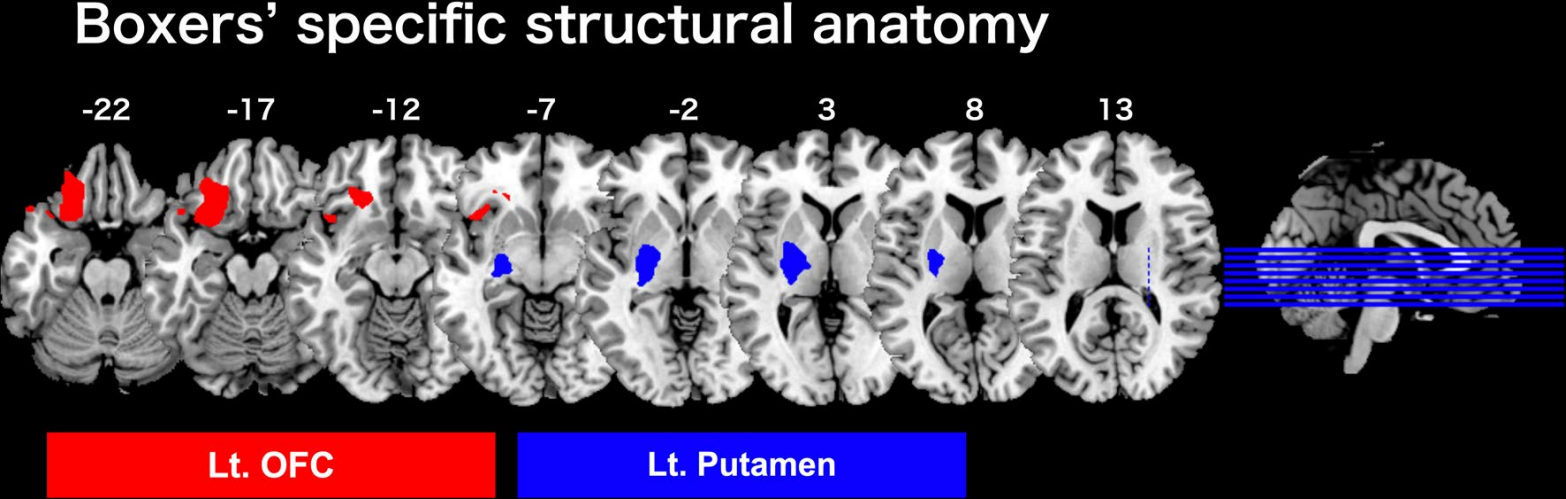
Boxers’ specific structural anatomy ([Boxers (Time1) > Controls]). The location of a significantly larger cluster in the left OFC and left putamen of boxers are shown as red and blue regions, respectively, on the z-axis. The statistical threshold for significant differences was set to FWE-corrected *p* < 0.05, at the cluster level, with uncorrected *p* < 0.001 at the voxel level. Lt, left; OFC, orbitofrontal cortex.

Compared with controls, boxers showed significantly larger GMV in the left putamen in the striatum [top peak = (− 22, − 18, − 5); cluster FWE-corrected *p* = 0.016; number of voxels = 1,299], and the left orbitofrontal cortex (OFC) [top peak = (21, 2, − 6); cluster FWE-corrected *p* < 0.001; number of voxels = 1,969] in contrast [Boxers (Time1) > Controls]. Otherwise, we did not find any significant clusters in the opposite contrast [Controls > Boxers (Time1)] (Fig. 3 and Table 2).

**Table 2:** Boxers specific anatomical structures ([Boxers (Time1) > Controls]).

	Cluster FWE *p*	Cluster size	t Value	Peak (x, y, z)
Lt. OFC	< 0.001	1969	5.80	(− 18, 29, − 20)
			5.65	(− 24, 27, − 18)
			5.63	(− 27, 36, − 21)
Lt. putamen	0.016	1299	5.55	(− 22, − 18, − 5)
			5.08	(− 27, − 26, − 2)

The statistical threshold for significant differences was set at FWE corrected *p* < 0.05, at the cluster level with uncorrected *p* < 0.001 at the voxel level.

Lt, left; OFC, orbitofrontal cortex.

### rs-fMRI results

**Figure 4:**
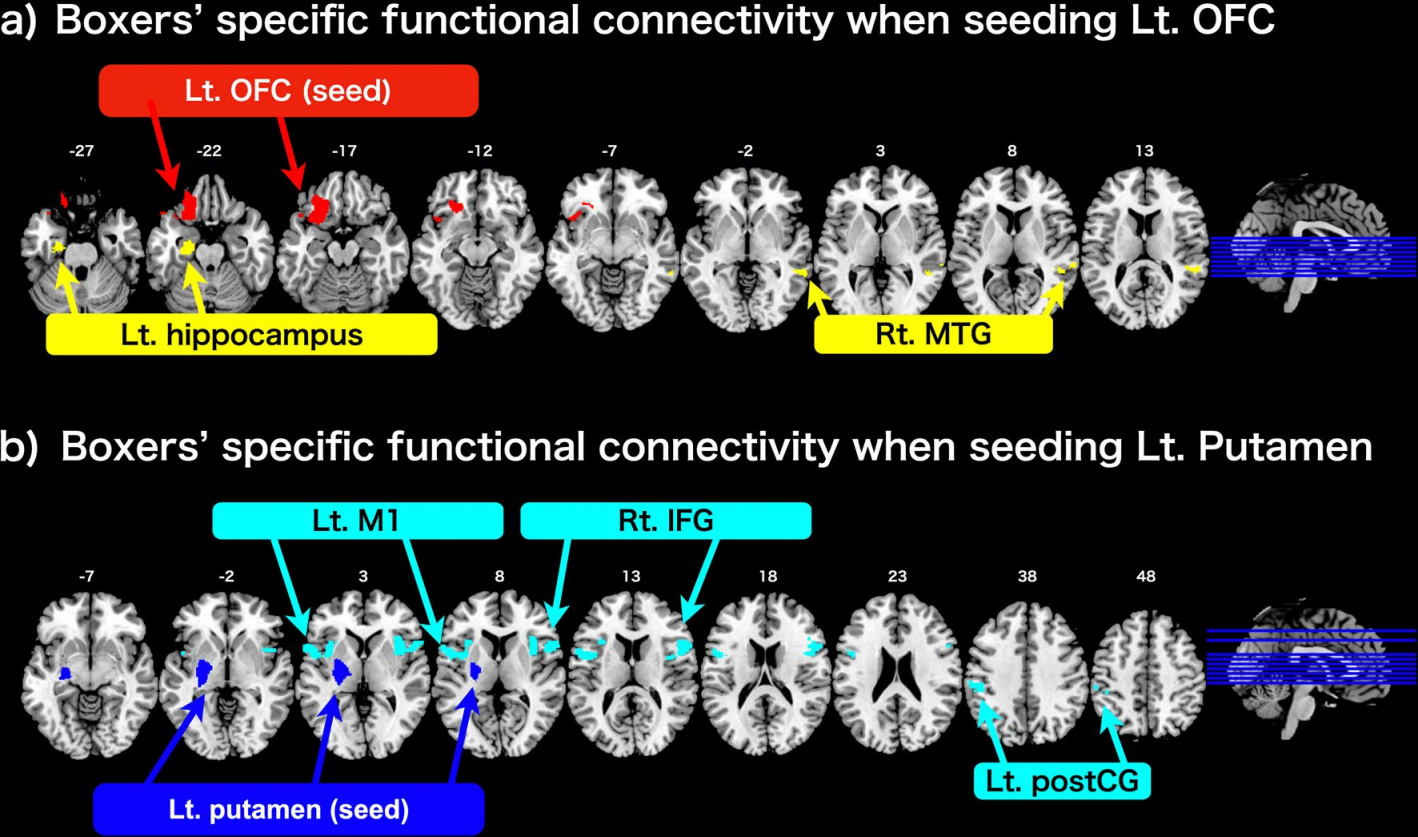
Boxers’ specific functional connectivity ([Boxers (Time1) > Controls]). (**a**) The statistical contrast between boxers and controls when seeding the left OFC (shown in red) delineated significant functional connectivity with the left hippocampus and the right MTG (shown in yellow colour respectively). (**b**) The statistical contrast between boxers and controls when seeding the left putamen (shown in blue) delineated significant functional connectivity with the left M1 cluster including the left premotor and anterior insular cortex, the left postCG, and Rt. IFG cluster including the right anterior insular cortex (shown in cyan colour respectively). The statistical threshold for significance was set at FWE-corrected *p* < 0.05 at the cluster level, with an uncorrected *p* < 0.001 at the voxel level. Lt, left; Rt, right; M1, primary motor cortex; MTG, middle temporal gyrus; postCG, postcentral gyrus; IFG, inferior frontal gyrus.

We compared the rs-fMRI data between boxers and controls to elucidate the functional connectivity specific to boxers. When seeding the left putamen cluster (Fig. 4b and Table 3), the contrast [Boxers [(Time1) > Controls] demonstrated a significant difference in functional connectivity between the left putamen (seed) and the following regions: the right inferior frontal gyrus (IFG) [top peak = (58, 14, 12); cluster FWE-corrected *p* < 0.001; number of voxels = 692], left primary motor cortex (M1) located in the precentral gyrus [top peak = (− 54, 4, 14); cluster FWE-corrected *p* < 0.001; number of voxels = 608], and left postcentral gyrus (postCG) [top peak = (− 42, − 38, 54); cluster FWE-corrected *p* = 0.006; number of voxels = 236]. When seeding the left OFC (Fig. 4a and Table 3), in the same contrast [Boxers [(Time1) > Controls], we found a significant difference in functional connectivity between the left OFC cluster (seed) and the following regions: the left hippocampus [top peak = (− 24, − 14, − 26); cluster FWE-corrected *p* = 0.033; number of voxels = 175] and the right middle temporal gyrus (MTG) [top peak = (60, − 40, − 4); cluster FWE-corrected *p* = 0.040; number of voxels = 168]. Otherwise, we did not find any significant difference in functional connectivity in the opposite contrast [Controls > Boxers (Time1)]. In addition, there was no significant difference in the functional connectivity in the contrasts [Boxers (Time2) > Boxers (Time1)] and [Boxers (Time2) > Boxers (Time3)], that is, no significant change in the functional connectivity in boxers was observed.

**Table 3:** Boxer specific functional connectivity ([Boxers (Time1) > Controls]).

	Cluster FWE *p*	Cluster size	t Value	Peak (x, y, z)
**Seed: Lt. OFC**				
Lt. hippocampus	0.033	175	5.05	(− 24, − 14, − 26)
			3.70	(− 26, − 24, − 20)
Rt. MTG	0.040	168	4.18	(60, − 40, − 4)
			4.07	(52, − 38, 12)
			3.90	(52, − 44, 2)
**Seed: Lt. putamen**				
Rt. IFG	< 0.001	692	6.97	(58, 14, 12)
			4.30	(50, 6, 14)
			4.06	(40, 10, 4)
Lt. M1	< 0.001	608	5.21	(− 54, 4, 14)
			4.06	(− 44, 4, 12)
			3.98	(− 58, 12, 4)
Lt. postCG	0.006	236	4.76	(− 42, − 38, 54)
			3.92	(− 48, − 30, 38)
			3.61	(− 56, − 28, 40)

The statistical threshold for significant differences was set at FWE corrected *p* < 0.05, at the cluster level with uncorrected *p* < 0.001 at the voxel level.

Lt, left; Rt, right; MTG, middle temporal gyrus; IFG, inferior frontal gyrus; M1, primary motor cortex; postCG, postcentral gyrus.

**Figure 5:**
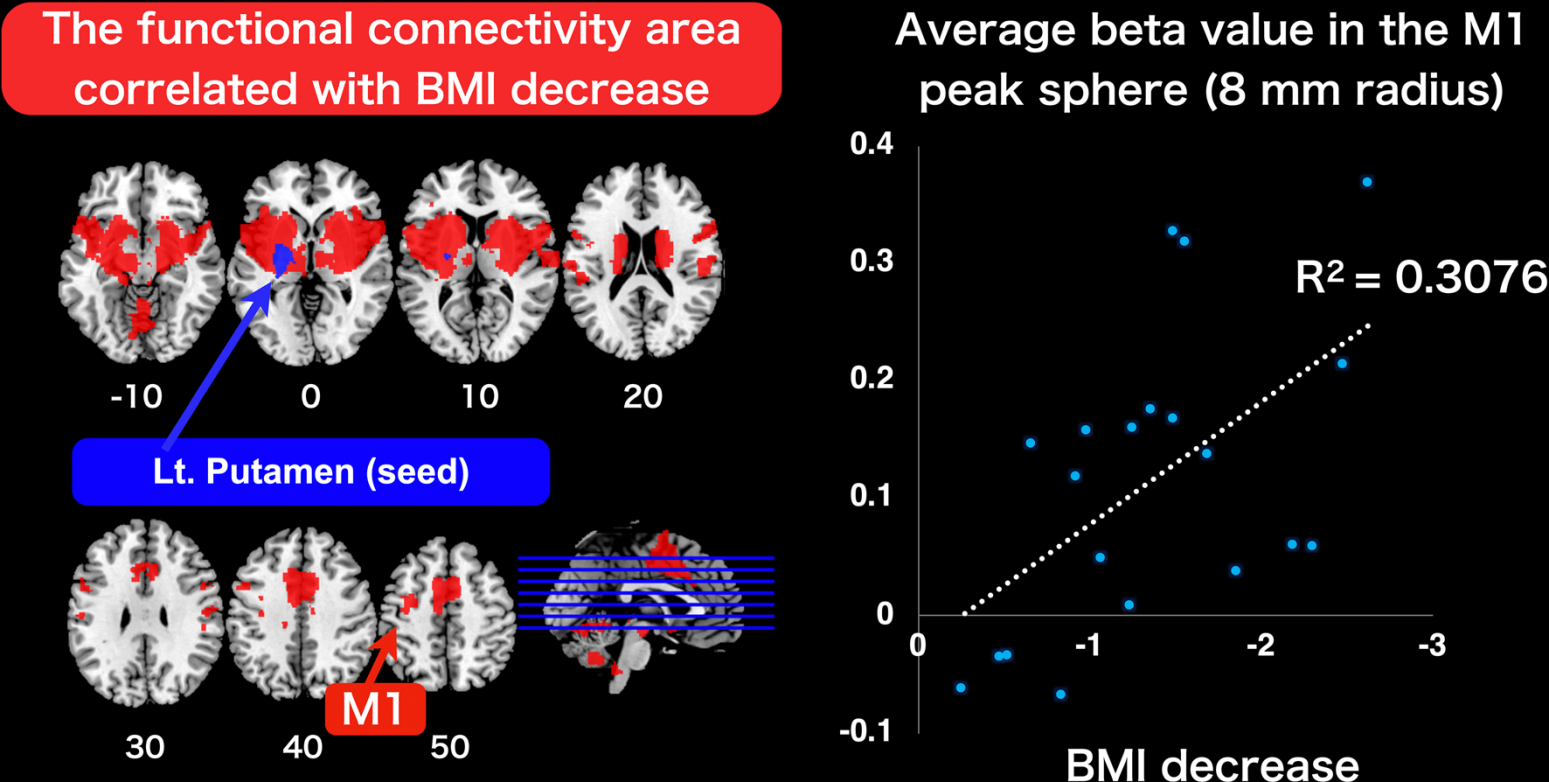
The functional connectivity area correlated with BMI decrease in Time2. The functional connectivity area (shown in red) that correlated with a decrease in BMI, seeding the left putamen cluster (shown in blue) at Time2, is shown. The M1 cluster is indicated by a red arrow. Using the eigenvariate function in SPM, we extracted the average beta value (as the functional connectivity value) in the sphere of an 8-mm radius (16-mm diameter) located at the peak of the left M1 cluster, based on our hypothesis testing the representation within the motor loop of boxers. The right scatter graph indicates a significant correlation (R^2^ = 0.3076, [*p* = 0.011]) between the decrease in BMI and the average beta value within the sphere of the left M1 cluster at Time2. BMI, body mass index; M1, primary motor cortex; SPM, statistical parametric mapping.

In a regression analysis between the BMI reduction data and functional connectivity data for seeding the putamen cluster at Time2, boxers showed significant BMI decreases covaried with functional connectivity between the putamen and brain regions, such as the left insula [top peak = (− 40, 2, 12); cluster FWE-corrected *p* < 0.001; number of voxels = 8897], right insula [top peak = (36, 6, 8); cluster FWE-corrected *p* < 0.001; number of voxels = 7927], cingulate gyrus (dorsal part of the anterior cingulate cortex) [top peak = (36, 6, 8); cluster FWE-corrected *p* < 0.001; number of voxels = 2463], and left M1 cluster [top peak = (− 30, − 4, 50); cluster FWE-corrected *p* = 0.002; number of voxels = 293) (Fig. 5 and Table 4).

**Table 4:** The functional connectivity area correlated with BMI decrease seeding the left putamen cluster at Time2.

	Cluster FWE *p*	Cluster size	*t* Value	Peak (x, y, z)
Lt. insula	< 0.001	8897	11.90	(− 40, 2, 12)
			9.23	(− 28, 12, 4)
			9.05	(− 38, 6, − 2)
Rt. insula	< 0.001	7927	8.42	(36, 6, 8)
			8.36	(62, 14, 18)
			7.94	(32, − 16, 8)
Cingulate gyrus (dorsal part of ACC)	< 0.001	2463	7.73	(− 10, 8, 48)
			7.28	(− 2, 6, 52)
			7.07	(− 6, 4, 40)
Lt. M1	0.002	293	6.67	(− 30, − 4, 50)
			6.04	(− 34, − 14, 46)
			5.27	(− 34, − 4, 42)

We present only the significant brain regions within the cerebral cortex (excluding the cerebellum and brainstem clusters for presentation purposes). The statistical threshold for significant differences was set at FWE corrected *p* < 0.05, at the cluster level with uncorrected *p* < 0.001 at the voxel level.

ACC, anterior cingulate cortex; BMI, body mass index; FWE, family-wise error; Lt, left; Rt, right; M1, primary motor cortex.

### DWI results

**Figure 6:**
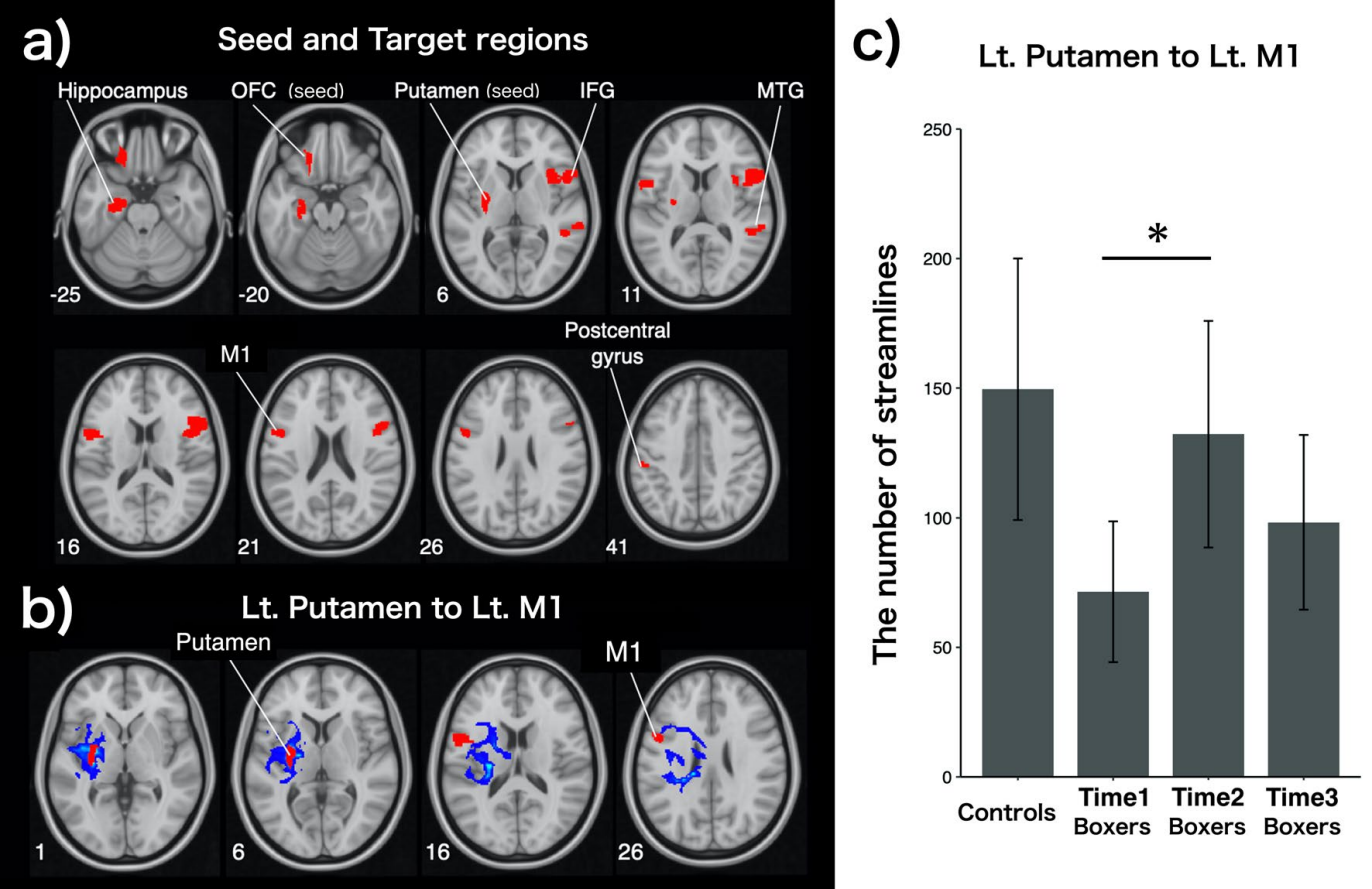
Diffusion tractography and the number of streamlines from the Lt. putamen to Lt. M1. (**a**) Seed and target brain regions used in diffusion tractography analysis are shown in red; These region-of-interests were defined based on the results from group-level analyses in the resting-state functional connectivity: from Lt. OFC (seed) to Lt. hippocampus and right MTG, and from Putamen (seed) to Lt. M1, Lt. postcentral gyrus and right IFG. **b**) Representative images of tractography results from the Lt. Putamen to Lt. M1 for a subject, are illustrated. Streamlines were originally drawn in native space, and then were transformed into MNI space. (**c**) The number of streamlines from the Lt. putamen to Lt. M1 in controls and boxers (Time1, Time2, and Time3). Only the number of streamlines from Lt. putamen to Lt. M1 between Time1 and Time2 in boxers shows a significant increase [*F*(2, 40) = 4.76, p = 0.02] in repeated measures ANOVA. Bonferroni correction was used in post-hoc analysis. Lt, left; OFC, orbitofrontal cortex; M1, primary motor cortex; MTG, middle temporal gyrus; IFG, inferior frontal gyrus; ANOVA, analysis of variance.

Based on the results of the functional connectivity analysis, we calculated the number of streamlines (structural connectivity strength) in controls and boxers (Time1, Time2, and Time3) from two seed regions (the left OFC or the left putamen) to the following five target regions (Fig. 6a): 1. the left hippocampus (from the left OFC), 2. the right MTG (from the left OFC), 3. the left postcentral gyrus (from the left putamen), 4. the left M1 (from the left putamen) and 5. the right IFG (from the left putamen). Figure 6b illustrates a representative image of tractography results from the left putamen to the left M1 for a subject, and Fig. 6c illustrates the number of streamlines from the left putamen to the left M1 in boxers (Time1, Time2, and Time3) and controls. A significant increase in the number of streamlines between the left M1 and the left putamen was found uniquely between Time1 and Time2 (Fig. 6c) [*F*(2, 40) = 4.76, *p* = 0.02] in repeated measures ANOVA. Bonferroni correction was used for the post-hoc analysis. No significant change in the number of streamlines in other contrasts was found.

## Discussion

Boxers showed enhanced structural connectivity between the M1 and putamen within the motor loop before the match (from Time1 to Time2). As discussed below, we suggest that the current findings may represent physically reinforced well-defined movements of the body of the boxer, induced by training with weight reduction for the match.

Boxing is a sport that comprises a wide variety of sequential movements, including offensive, defensive, and counterattack skills^14^. The M1 (located in the precentral gyrus) is the primary site for movement generation^22,23^. Functional and structural changes occur in M1 during motor skill training^17^. For example, rigorous motor skill training in athletes induces an expansion of the proximal muscle representation in the M1 contralateral to the dominant arm^24^. The smoothness of the movement is represented by the neuronal activity in the motor loop^15,16^. M1 plays an especially important role in the smoothness of movement, as well as in the acquisition and optimization of a novel series of inter-related movements induced by repeated motor skill training^13,25^. Such well-trained smooth movement is accurate and well-defined, leading to performance improvement^13^.

We first showed that one of the specifically altered brain structures in boxers was the left putamen (Fig. 3). The putamen of the basal ganglia is the main corticostriatal input station^26^. Neurophysiological studies have identified the putamen, together with the M1 and cerebellum, to be the major brain regions involved in motor skill learning^26^. The essential brain module dedicated to motor sequence learning resides in the putamen^13^. Together with a previous finding that increased GMV in the putamen was accompanied by repetitive motor skill training in athletes^27^, it is conceivable that boxers’ higher GMV in the putamen, relative to controls, would be the result of repeated motor skill training before a match.

Boxers exhibited significantly enhanced structural connectivity between the M1 and putamen within the motor loop before the match (from Time1 to Time2) (Fig. 6). The putamen exhibits a somatotopic organization from the forelimb region of the M1 in monkeys, indicating functional and structural connectivity between the M1 and putamen within the motor loop^28^. Doyon et al.^29^ reviewed human functional neuroimaging studies that demonstrated that as motor skill training gradually progresses and consolidates, the contribution between the M1 and putamen progressively increases and enables sequential movements, indicating that the connectivity between the M1 and putamen is crucial for establishing the well-defined physical movements induced by motor skill training. Larger white matter volume has often been associated with higher motor skill training^30^. Thus, we suggest that the enhanced structural connectivity in the white matter before the match is a representation of the well-defined movements of the body induced by motor skill training for the match in boxers.

In addition, we found that controls (non-athletes who did not undergo daily training) had equivalent numbers of streamlines from the left putamen to Lt. M1 as boxers did at Time2 (Fig. 6b). In this regard, one review^30^ described the interpretation of white matter plasticity findings in elite athletes and controls as “challenging.” For example, compared to controls, professional ballet dancers have been found to have lower white matter volume and lower fractional anisotropy underlying motor skill training^31^. Variables such as the sports discipline in question, the amount of training, training content, training stage, or strategy might underlie the discrepancies among the studies^30^. Histologically, de novo myelination in the white matter with long timescales, such as weeks or months, has been found to be important for motor skill training^32,33^. Therefore, longitudinal observation in such timescales is a crucial factor for assessing white matter plasticity using MRI measures^30^, as we present here in boxers.

Sensorimotor coordination in boxers is also worth discussing. When training a new motor sequence, we must execute the correct order of movements while simultaneously optimizing sensorimotor parameters, such as trajectory, timing, velocity, and force, otherwise known as sensorimotor coordination^17,26^. In the current rs-fMRI results, the M1 cluster contains activation not only in the precentral gyrus but also in the premotor cortex and anterior insular cortex (Fig. 4b). Although we labeled the identified cluster “M1” because the cluster contains the most active voxels, in the precentral gyrus, the peak coordinates (− 54, 4, 14) in the “M1” cluster (Table 3) reside in the ventral premotor cortex^34^. The ventral premotor cortex is directly connected to the precentral gyrus and receives rich input from the second somatosensory area and the anterior insular cortex^35^. This area is also known to be important for sensorimotor coordination, especially visuo-motor coordination^35,36^. In monkeys, electrical stimulation of this area causes an apparent defensive movement as if protecting the body, suggesting that the ventral premotor region may play a role in maintaining a safety margin around the body and guiding the movement in response to nearby objects with sensory-motor coordination^37^. In addition, activation in the precentral gyrus in the M1 cluster was located dorsal to that of the ventral premotor cortex (Fig. 4b). From a traditional somatotopic view of the human M1, the current precentral activation corresponds to the lip, tongue, and face as ventral sensorimotor cortex^38^ or borderline hand^39^. However, such a somatotopic map in the M1 becomes more overlapping as we learn coordinated movements, suggesting that the M1 may participate in integrating muscles in meaningful ways rather than in segregating the control of individual muscles^40,41^. In a mapping study, polysensory neurons, which respond to tactile, visual, and auditory stimuli, were found to be clustered in the precentral gyrus, dorsal to the ventral premotor cortex (the same position as that of current precentral activation in the M1 cluster), suggesting that the current zone in the precentral gyrus plays an important role in sensorimotor coordination^42^. Additionally, electrical stimulation of this precentral zone evokes a specific set of movements typically used to defend the body from objects that are near approaching^42^. Sensorimotor coordination and defensive movements are required for boxer training. Therefore, we consider that the current activation in the M1 cluster represents activation associated with sensorimotor coordination, especially with defensive movements in boxers before the match, rather than the somatotopic representation of the body parts.

In contrast to the enhanced structural connectivity observed in boxers in this study, we could not find any functionally corresponding enhanced changes between the M1 cluster and putamen from Time1 to Time2. Boxers instead showed a correlation between BMI reduction and average beta value within M1 in the functional connectivity area, seeding the putamen at Time2 (within 1 week prior to a match; Fig. 5). These findings imply that the functional connectivity between the M1 cluster and putamen in boxers was associated with training and weight reduction before the match.

It is important to discuss another specific finding in boxers: the OFC (Fig. 3). The OFC has been regarded as the strongest brain region linking food intake, appetite, and other types of rewards and hedonic experiences^43^. In humans and higher primates, the OFC receives multimodal information about the sensory properties of food, representing incentive salience, hedonic impact, and subjective hedonic experience^44^. The subjective pleasantness of food is represented in the OFC, together with the reward value of taste, olfactory, and somatosensory components of food^44^. Similarly, the OFC encodes food reward value only when hunger is present^45^. Body mass regulation in boxing creates a number of unique challenges, and practices related to the manipulation of body mass before the match must be integrated into diet strategies and performance considerations^11^. Boxers typically reduce their weight by continuous physical training and restricting oral intake in both the short- and long-term, prior to the weigh-in^9–11^. The boxer’s typical approach to weight reduction usually results in a 5–6 kg average weight reduction via severe acute and chronic energy restriction and dehydration^46^. Hunger is the most frequently mentioned claim during weight reduction^47^. In the current rs-fMRI results, the left OFC of boxers showed significant functional connectivity with the left hippocampus and right MTG (Table 3). The hippocampus is not only central to memory but is also involved in appetite by monitoring the state of hunger and satiety in humans^48^. The MTG also has a functional interaction with the OFC in long-term memory, especially with regard to food stimuli^49,50^. Therefore, we suppose that the higher GMV in OFC than controls might represent the strong hunger and food craving observed during the weight reduction period in boxers, and boxers’ OFC might have functional connectivity with the regions associated with appetite and food memory.

Future investigations into the physical and physiological attributes of boxers are required to enrich the current data set. Because the strategies before a match in combat sports are generally associated with multiple factors, such as diet, training strategies, and time management^10^, the relationship between weight reduction before the match and athletes’ performance improvement would be qualitative assessments. Nevertheless, future studies may require real quantitative performance values and psychological values, such as hunger or distress before the match, as well as event-related potentials in a go/no-go task of professional fencers and boxers^51^, hand speed measurement in boxers^52^, or reaction time and punch analysis in boxers^53^. Such measurements could enable us to validate the correlations between these physiological performance values (improvement) and brain plasticity in athletes.

In conclusion, boxers showed enhanced structural connectivity between the M1 and putamen within the motor loop before the match. We suggest that the current findings might represent physically reinforced well-defined movements of the body of the boxer, induced by training with weight reduction for the match. The current finding might provide a newly grounded significance of the training accompanying weight reduction before a match for a combat sports match.

## Methods

### General design and participants

A cross-sectional and longitudinal approach was used to test our hypothesis, as shown in Fig. 1. Two groups of participants (boxers and controls) were recruited. The boxer group consisted of 21 male professional licensed boxers from the Japanese Boxing Commission (aged 26.7 ± 4.0 years), who had experienced 13.4 ± 8.9 professional matches before enrolment in this study (data are presented as means ± standard deviation, unless otherwise indicated). The control group consisted of 22 healthy non-athletic males (aged 27.2 ± 3.8 years). Controls were age-, sex-, and BMI-matched with boxers. These inclusion criteria for controls were based on previous studies in the sports domain, especially after findings showing that BMI correlates with regional morphology^54,55^. Boxers underwent MRI scanning at three time points for longitudinal investigation: Time1 (1 month before the match), Time2 (within 1 week before the match), and Time3 (1 month after the match), while controls underwent scanning only once. The Time3 measurements were conducted to explore whether the boxer’s BMI would reverse after the match as an indicator of their training load after the match, based on the results of a previous study on marathon runners^56^.

All participants were right-handed according to the Edinburgh Handedness Inventory^57^, with no history of psychiatric or neurological disorders. All participants received monetary compensation for their time. The protocol was approved by the ethical committee of Teikyo University, Tokyo, Japan (UMIN000017635). All participants completed the Stanford Sleepiness Scale, which examines drowsiness before MRI scanning (a seven-grade evaluation)^58^. All participants provided written informed consent and underwent weight and height measurements immediately before MRI scanning.

### Imaging measurement protocol

All MRI data were acquired using a 3 T scanner (MAGNETOM Skyra; Siemens, Ltd., Erlangen, Germany) at Teikyo University. Each participant's head was immobilized within a 20-channel phased-array head coil. The total experiment time was approximately 45 min, including body weight and height measurements, and the MRI scan (total imaging acquisition time was 30 min 22 s).

#### VBM

Whole-brain high-resolution T1-weighted anatomical MRI scan using a magnetization prepared rapid acquisition gradient echo (MP-RAGE) was conducted on each participant (echo time [TE] = 2.28 ms; repetition time [TR] = 2,300 ms; field of view [FOV] = 256 mm × 256 mm; flip angle [FA] = 9°; matrix size = 1 mm × 1 mm × 1 mm; slice thickness = 1 mm; and slice numbers = 240 coronal slices).

##### rs-fMRI

rs-fMRI scans were obtained while participants were resting comfortably in the scanner with their eyes open for a period of 6 min 15 s, using an echo planar imaging (EPI) gradient-echo sequence (TE = 30 ms; TR = 2,500 ms; FOV = 212 mm^2^ × 212 mm^2^; FA = 80°; matrix size = 3.31 mm × 3.31 mm × 4 mm; 40 slices; slice thickness = 3.2 mm; total number of volumes = 148).

#### DWI

Diffusion-weighted spin echo EPI sequence scans were obtained with 2.0 mm slice thickness and a reconstructed image matrix = 1 mm × 1 mm × 2 mm (TE = 71 ms, TR = 10,400 ms, FOV = 220 mm × 220 mm, FA = 90°, b-value = 1,000 s/mm^2^, bandwidth = 1818 Hz/pixel; slice numbers = 80 transverse slices without inter-slice gap, covering the whole brain). Diffusion was measured in 30 non-collinear directions with a b-value of 1000 s/mm^2^, followed by a non-diffusion-weighted volume (reference volume).

### Data processing and statistical analyses

#### VBM

For voxel-based morphometry, the VBM8 toolbox (revision 435) implemented in Statistical Parametric Mapping (SPM) 8 (revision 5236; The Wellcome Trust Centre for Neuroimaging; www.fil.ion.ucl.ac.uk/spm) was applied^20,59^. Structural images were corrected for bias-field inhomogeneity and spatially normalized with diffeomorphic anatomical registration through exponentiated Lie algebra (DARTEL) to the Montreal Neurological Institute (MNI) template. Tissues were classified as gray matter, white matter, or cerebrospinal fluid. In the modulation process, nonlinear deformation was used for normalization so that voxel intensities reflected regional GMVs adjusted for individual brain sizes. Images were then smoothed to a Gaussian kernel with a full width at half maximum (FWHM) of 8 mm.

After pre-processing the structural images, we conducted a group analysis. We further conducted a whole-brain analysis to investigate GMV differences between the groups using a two-sample t-test, using age as the 'effects of no interest.' The statistical threshold of significant difference was set at the family-wise error (FWE) corrected *p* < 0.05 at the cluster level, with uncorrected *p* < 0.001 as a cluster-forming threshold using the nonstationary correction^60^. The clusters identified in the group analysis were labeled using the Talairach daemon^61^ after accounting for the discrepancy between the MNI space and Talairach space^62^. Significant clusters specific to boxers were revealed by the contrasts [Boxers (Time1) > Controls] and [Controls > Boxers (Time1)] were defined as the seed regions for the following longitudinal connectivity analysis.

##### rs-fMRI

The functional images were pre-processed using SPM8 (revision 5236; The Wellcome Trust Centre for Neuroimaging). The first four volumes of each resting-state fMRI run were discarded to allow the blood-oxygen-level-dependent (BOLD) signal to reach a steady state. After performing motion correction, we used Fourier phase-shift interpolation to correct the slice timing of each image to the middle slice. The mean of the realigned EPI images was then co-registered with the T1-weighted MP-RAGE image. Subsequently, the co-registered T1-weighted MP-RAGE image was normalized to the MNI template using linear and nonlinear three-dimensional transformations. The parameters from this normalization process were applied to each EPI image. Finally, the anatomically normalized EPI images were resampled to a voxel size of 2 mm × 2 mm × 2 mm and spatially smoothed using a Gaussian kernel of 8 mm FWHM. After realignment, we examined head movement parameters.

After pre-processing the functional images, we conducted a functional connectivity analysis using the CONN toolbox (version 15.h) on SPM8. In the functional connectivity analysis, we set the clusters as seed regions based on the cross-sectional VBM analysis above. Using the CONN toolbox, we conducted a seed-driven functional connectivity analysis (seed-to-voxel analysis) in which Pearson's correlation coefficient was calculated between the seed time course and the time course of all other voxels^63^. The correlation coefficients were then converted to normally distributed scores using Fisher's transformation to allow for general linear model analysis, using the same significance threshold (FWE corrected *p* < 0.05 at the cluster level, with uncorrected *p* < 0.001 at the voxel level). Differences between groups were assessed using t-tests. To elucidate the functional connectivity specific to boxers, we compared boxers with controls [Boxers (Time1) > Controls] and [Controls > Boxers (Time1)]. To investigate enhanced functional connectivity within the motor loop before the match, we conducted the following contrasts: [boxers (Time2) > boxes (Time1)] and [boxes (Time2) > boxes (Time3)]. Furthermore, to investigate the effect of weight reduction on functional connectivity at the time point before the match (Time2), we also conducted a regression analysis using the BMI reduction data ([boxers’ BMI in Time2] minus [boxers’ BMI in Time1]) and Time2 functional connectivity data. In the regression analysis, we defined BMI decrease as an effect of interest, sex, and age as the effects of no interest. The clusters identified were labeled using automated anatomical labeling (AAL) atlases^64^ implemented in CONN. We adopted the label of the AAL atlas, whose region was the most covered with voxels in the identified cluster. This labeling procedure is because the cluster correction we adopted (FWE-corrected *p* < 0.05 at the cluster level) measures in units of contiguous voxels and is determined based on the estimated distribution of cluster sizes, rather than the location of the peak coordinate^65^.

#### DWI

Individual DWI data were pre-processed using the Diffusion Toolbox in the FMRIB Software Library (FSL: www.fmrib.ox.ac.uk/analysis)^66,67^. Motion and eddy-current corrections were performed using affine registration of the b0 images. The skull and non-brain tissues were removed using the brain extraction tool. Probability density functions for up to two principal fiber directions were estimated at each voxel in the brain using the Bayesian estimation of diffusion parameters that were obtained via a sampling technique toolbox (BedpostX)^68^. Finally, to illustrate the streamlines from the seed to the target region, we performed multi-fiber probabilistic tractography using ProbtrackX implemented in FSL (maximum number of steps = 2,000, curvature threshold = 0.2, step length = 0.5). We drew 5,000 streamlines from each voxel in each seed region to the target regions. We defined the seed and target regions based on the results of functional connectivity analysis^69^.

To compare the number of streamlines in each white matter pathway between groups or times, we defined the mask for each white matter pathway based on the individual streamline maps from each seed to the target region. First, the individual tract was defined as the voxels where the number of passing streamlines was above 100 and was binarized. Second, we defined group-level masks of each tract as voxels that were included in the individual tract from half of the subjects in each group. Then, the mask for each tract was defined as the shared voxels between the two group-level tracts (i.e., the boxer’s tract and control’s tract). Finally, the number of streamlines was estimated by averaging the number within each mask of the tracts in each individual and at each time point.

### Ethics approval

The protocol was approved by the ethical committee of Teikyo University, Tokyo, Japan (UMIN000017635).

## Data Availability

Available from the corresponding author upon reasonable request.

## References

[1] Pascual-Leone A, Amedi A, Fregni F, Merabet LB (2005). The plastic human brain cortex. Annu. Rev. Neurosci..

[2] May A (2011). Experience-dependent structural plasticity in the adult human brain. Trends Cogn. Sci..

[3] Zatorre RJ, Fields RD, Johansen-Berg H (2012). Plasticity in gray and white: Neuroimaging changes in brain structure during learning. Nat. Neurosci..

[4] Yarrow K, Brown P, Krakauer JW (2009). Inside the brain of an elite athlete: The neural processes that support high achievement in sports. Nat. Rev. Neurosci..

[5] Makris S (2014). Sport neuroscience revisited (?): A commentary. Front. Hum. Neurosci..

[6] Nakata H, Yoshie M, Miura A, Kudo K (2010). Characteristics of the athletes' brain: Evidence from neurophysiology and neuroimaging. Brain Res. Rev..

[7] Chang Y (2014). Reorganization and plastic changes of the human brain associated with skill learning and expertise. Front. Hum. Neurosci..

[8] Draganski B (2004). Neuroplasticity: Changes in grey matter induced by training. Nature.

[9] Craig AH (2009). Making Weight in Combat Sports. Combat Sports Medicine Ch.

[10] Franchini E, Brito CJ, Artioli GG (2012). Weight loss in combat sports: Physiological, psychological and performance effects. J. Int. Soc. Sports. Nutr..

[11] Reale R, Slater G, Burke LM (2017). Acute-weight-loss strategies for combat sports and applications to Olympic success. Int. J. Sports. Physiol. Perform..

[12] Chaabène H (2015). Amateur boxing: Physical and physiological attributes. Sports Med..

[13] Orban P (2010). The multifaceted nature of the relationship between performance and brain activity in motor sequence learning. Neuroimage.

[14] Ashker EIS (2012). Technical performance effectiveness subsequent to complex motor skills training in young boxers. Eur. J. Sport Sci..

[15] Nambu A (2004). A new dynamic model of the cortico-basal ganglia loop. Prog. Brain Res..

[16] Middleton FA, Strick PL (2000). Basal ganglia and cerebellar loops: Motor and cognitive circuits. Brain Res. Rev..

[17] Hikosaka O, Nakamura K, Sakai K, Nakahara H (2002). Central mechanisms of motor skill learning. Curr. Opin. Neurobiol..

[18] Doyon J (2009). Contributions of the basal ganglia and functionally related brain structures to motor learning. Behav. Brain Res..

[19] Lakhani B (2016). Motor skill acquisition promotes human brain myelin plasticity. Neural Plast..

[20] Ashburner J, Friston KJ (2000). Voxel-based morphometry–the methods. Neuroimage.

[21] Fox MD, Zhang D, Snyder AZ, Raichle ME (2009). The global signal and observed anticorrelated resting state brain networks. J. Neurophysiol..

[22] Omrani M, Kaufman MT, Hatsopoulos NG, Cheney PD (2017). Perspectives on classical controversies about the motor cortex. J Neurophysiol..

[23] Banker L, Tadi P (2020). Neuroanatomy, Precentral Gyrus.

[24] Tyc F, Boyadjian A, Devanne H (2005). Motor cortex plasticity induced by extensive training revealed by transcranial magnetic stimulation in human. Eur. J. Neurosci..

[25] Shibasaki H (1993). Both primary motor cortex and supplementary motor area play an important role in complex finger movement. Brain.

[26] Penhune VB, Steele CJ (2012). Parallel contributions of cerebellar, striatal and M1 mechanisms to motor sequence learning. Behav. Brain Res..

[27] Park IS (2011). Basketball training increases striatum volume. Hum. Mov. Sci..

[28] Nambu A, Kaneda K, Tokuno H, Takada M (2002). Organization of corticostriatal motor inputs in monkey putamen. J. Neurophysiol..

[29] Doyon J, Gabitov E, Vahdat S, Lungu O, Boutin A (2018). Current issues related to motor sequence learning in humans. Curr. Opin. Behav. Sci..

[30] Sampaio-Baptista C, Johansen-Berg H (2017). White matter plasticity in the adult brain. Neuron.

[31] Hänggi J, Koeneke S, Bezzola L, Jäncke L (2010). Structural neuroplasticity in the sensorimotor network of professional female ballet dancers. Hum. Brain Mapp..

[32] McKenzie IA (2014). Motor skill learning requires active central myelination. Science.

[33] Xiao L (2016). Rapid production of new oligodendrocytes is required in the earliest stages of motor-skill learning. Nat. Neurosci..

[34] Mayka MA, Corcos DM, Leurgans SE, Vaillancourt DE (2006). Three-dimensional locations and boundaries of motor and premotor cortices as defined by functional brain imaging: A meta-analysis. Neuroimage.

[35] Binkofski F, Buccino G (2006). The role of ventral premotor cortex in action execution and action understanding. J. Physiol..

[36] Picard N, Strick PL (2001). Imaging the premotor areas. Curr. Opin. Neurobiol..

[37] Graziano MS, Cooke DF (2006). Parieto-frontal interactions, personal space, and defensive behavior. Neuropsychologia.

[38] Conant D, Bouchard KE, Chang EF (2014). Speech map in the human ventral sensory-motor cortex. Curr. Opin. Neurobiol..

[39] Meier JD, Aflalo TN, Kastner S, Graziano MS (2008). Complex organization of human primary motor cortex: A high-resolution fMRI study. J. Neurophysiol..

[40] Graziano MS, Aflalo TN (2007). Mapping behavioral repertoire onto the cortex. Neuron.

[41] Harrison TC, Murphy TH (2014). Motor maps and the cortical control of movement. Curr. Opin. Neurobiol..

[42] Cooke DF, Graziano MS (2004). Sensorimotor integration in the precentral gyrus: Polysensory neurons and defensive movements. J. Neurophysiol..

[43] Kringelbach ML (2005). The human orbitofrontal cortex: Linking reward to hedonic experience. Nat. Rev. Neurosci..

[44] Kringelbach ML, O'Doherty J, Rolls ET, Andrews C (2003). Activation of the human orbitofrontal cortex to a liquid food stimulus is correlated with its subjective pleasantness. Cereb. Cortex.

[45] Rolls ET (2016). Reward systems in the brain and nutrition. Annu. Rev. Nutr..

[46] Morton JP, Robertson C, Sutton L, MacLaren DP (2010). Making the weight: A case study from professional boxing. Int. J. Sport Nutr. Exerc. Metab..

[47] Pettersson S, Pipping EM, Berg CM (2012). The food and weight combat. A problematic fight for the elite combat sports athlete. Appetite.

[48] Volkow ND, Wang GJ, Baler RD (2011). Reward, dopamine and the control of food intake: Implications for obesity. Trends Cogn. Sci..

[49] Simons JS, Spiers HJ (2003). Prefrontal and medial temporal lobe interactions in long-term memory. Nat. Rev. Neurosci..

[50] Simmons WK, Martin A, Barsalou LW (2005). Pictures of appetizing foods activate gustatory cortices for taste and reward. Cereb. Cortex.

[51] Bianco V, Di Russo F, Perri RL, Berchicci M (2017). Different proactive and reactive action control in fencers' and boxers' brain. Neuroscience.

[52] Kimm D, Thiel DV (2015). Hand speed measurements in boxing. Procedia Eng..

[53] Chadli S, Ababou N, Ababou A (2014). A new instrument for punch analysis in boxing. Procedia Eng..

[54] Bobb JF, Schwartz BS, Davatzikos C, Caffo B (2014). Cross-sectional and longitudinal association of body mass index and brain volume. Hum. Brain Mapp..

[55] Schlaffke L (2014). Sports and brain morphology—A voxel-based morphometry study with endurance athletes and martial artists. Neuroscience.

[56] Freund W (2014). Regionally accentuated reversible brain grey matter reduction in ultra marathon runners detected by voxel-based morphometry. BMC Sports Sci. Med. Rehabil..

[57] Oldfield RC (1971). The assessment and analysis of handedness: The Edinburgh Inventory. Neuropsychologia.

[58] Hoddes E, Dement WZV (1972). The development and use of the Stanford Sleepiness Scale (SSS). Psychophysiology.

[59] Ashburner J (2007). A fast diffeomorphic image registration algorithm. Neuroimage.

[60] Hayasaka S, Phan KL, Liberzon I, Worsley KJ, Nichols TE (2004). Nonstationary cluster-size inference with random field and permutation methods. Neuroimage.

[61] Lancaster JL (2000). Automated Talairach atlas labels for functional brain mapping. Hum. Brain Mapp..

[62] Lancaster JL (2007). Bias between MNI and Talairach coordinates analyzed using the ICBM-152 brain template. Hum. Brain Mapp..

[63] Whitfield-Gabrieli S, Nieto-Castanon A (2012). Conn: A functional connectivity toolbox for correlated and anticorrelated brain networks. Brain Connect..

[64] Tzourio-Mazoyer N (2002). Automated anatomical labeling of activations in SPM using a macroscopic anatomical parcellation of the MNI MRI single-subject brain. Neuroimage.

[65] Friston KJ, Worsley KJ, Frackowiak RS, Mazziotta JC, Evans AC (1994). Assessing the significance of focal activations using their spatial extent. Hum. Brain Mapp..

[66] Smith SM (2004). Advances in functional and structural MR image analysis and implementation as FSL. Neuroimage.

[67] Woolrich MW (2009). Bayesian analysis of neuroimaging data in FSL. Neuroimage.

[68] Behrens TE, Berg HJ, Jbabdi S, Rushworth MF, Woolrich MW (2007). Probabilistic diffusion tractography with multiple fibre orientations: What can we gain?. Neuroimage.

[69] van den Heuvel MP, Mandl RC, Kahn RS, Hulshoff Pol HE (2009). Functionally linked resting-state networks reflect the underlying structural connectivity architecture of the human brain. Hum. Brain Mapp..

